# Potential effect of household environment on prevalence of tuberculosis in India: evidence from the recent round of a cross-sectional survey

**DOI:** 10.1186/s12890-018-0627-3

**Published:** 2018-05-03

**Authors:** S. K. Singh, Gyan Chandra Kashyap, Parul Puri

**Affiliations:** 1 Department of Mathematical Demography & Statistics, International Institute for Population Sciences, Govandi Station Road, Deonar Mumbai, 400088 India; 2 International Institute for Population Sciences, Govandi Station Road, Deonar Mumbai, 400088 India

**Keywords:** Household, Environment, Prevalence of Tuberculosis, India

## Abstract

**Background:**

Tuberculosis (TB) has been a major health problem globally since ages, and even today, it is a major cause of morbidity in millions of people each year. In 2015 alone, TB accounted for about 1.4 million deaths globally, with India carrying the biggest burden of the disease. The physical environment of the household, an individual living in, has a significant influence on the incidence of TB. Thus, an understanding of the socio-economic, demographic and environmental factors that individuals are exposed to is of importance. The objective of present study is to examine the association of household environment with the prevalence of Tuberculosis in India.

**Methods:**

The study utilizes data from the fourth round of National Family Health Survey (NFHS-4), 2015-16, which was collected from self-reported information pertaining to Tuberculosis in the household questionnaire. The specific question was, “Does any usual resident of your household suffer from tuberculosis?” the response to which helped in the detection of Tuberculosis. Binary Logistic regression was performed from which appropriate inferences are drawn on the association of household environment with Tuberculosis.

**Results:**

Prevalence of TB was found to be the highest among elderly people (0.9%), no education (0.4%) and people belonging to the poorest wealth quintile (0.53%). Family members who were regularly (daily) exposed to smoke (second-hand smoke) inside the house were more prone to getting tuberculosis (OR = 1.49; CI = 1.39-1.61) as compared with households where people do not smoke inside the house. Further, households having a finished wall (OR = 0.7; CI = 0.6-0.8) are less likely to get TB than the households with mud walls. Households that shared their toilets with other households are more likely to get hold of Tuberculosis (OR = 1.2; CI = 1.1-1.4).

**Conclusions:**

Results strongly suggest that a contaminated household environment increases the risk of tuberculosis in India. There are multiple risk factors that are strongly associated with Tuberculosis: smoke inside house, type of cooking fuel, separate kitchen, floor, roofing and wall material, number of persons sleeping in a room, sharing toilet and potable water with other households; and individual characteristics such as age, sex, educational attainment, marital status, place of residence and wealth index.

**Electronic supplementary material:**

The online version of this article (10.1186/s12890-018-0627-3) contains supplementary material, which is available to authorized users.

## Background

The Sustainable Development Goals (SDG, 2015) are aimed at building an inclusive, sustainable and resilient future by incorporating economic, social and environmental protection for people and planet in order to ensure good health and well-being for all [1]. “The future will either be green or not at all”, said Australian environmentalist Robert James “Bob” Brown. A clean environment is mandatory for human health and well-being.

The best-known health impacts are related to environmental (air) pollution, poor water quality, and inadequate sanitation. Air pollution is an adulteration of the environment by any chemical, physical or biological agent that modifies the conventional characteristics of the atmosphere [2]. Owing to the increasing trends in the number of motor vehicles and industries, it is commonly believed that the problem is associated with developed urban areas. In reality, however, air pollution tends to be higher in developing regions. This is attributable to the combustion of biomass fuel indoors [3]. Indoor air pollution is not only dependent on the combustion of solid fuel, but it is also affected by overcrowded housing conditions and exposure to second-hand smoke inside the house.

Globally, around three billion people use solid fuel like wood, animal dung, crop waste and coal for cooking and keeping their houses warm. These fuels have low combustion efficiency and highly polluting emissions. Biomass smoke contains many harmful components, including suspended particulate matter, nitrogen oxides, carbon monoxide, etc. Regular exposure to these air pollutants can cause serious health problems [4–6]. Estimates suggest that over four million people die prematurely from illnesses attributable to the household air pollution resulting from cooking with solid fuels. In India, estimates from the National Family Health Survey (NFHS-4) suggest that about 55% of the household use solid fuel for cooking, among which about 99% use open fire/*chullah* for cooking. About 41% of household uses wood as their primary cooking fuel. Further, about half of all households do not have a separate kitchen [7].

Tuberculosis (TB) has been a major health problem globally since ages, and even today, it is a major cause of morbidity in millions of people each year. Tuberculosis (TB) is ranked among the top ten causes of deaths worldwide and as one of the leading causes of death from infectious disease. In 2015 alone, TB accounted for about 1.4 million deaths globally with India carrying the biggest burden of the disease [8]. The proportion of TB patients is higher in India because of poor socio-economic and environmental conditions. According to NFHS-4, the prevalence of TB is 316 per 100,000 persons in India [7].

The Indian government has envisaged a “TB-free India” by 2017, aimed at reducing the burden of TB in Indian sub-continent [9]. Studies show indoor air pollution as a significant risk factor for the occurrence of TB. However, the findings of most studies are based on small study populations [10, 11]. Existing literature suggests a number of factors associated with TB infection, including demographic, socio-economic and environmental factors, such as age, sex, level of education, marital status, place of residence, wealth, overcrowding, poor housing and household environment factors [3, 12–14].

The studies also highlight the higher prevalence of TB in men [14, 15], elderly, poor and malnourished [14], those belonging to urban areas, living in *kuchha* (usually made of mud) house and other environmental factors [10, 16]. About 26% of the cases of TB is attributed to Indoor Pollution [17]. However, the nature of the relationship between indoor air pollution and tuberculosis has not been in much focus. By considering the exposure to all environmental factors present in households, the present study tries to identify the potential household environmental factors which enhance the risk of prevalence of Tuberculosis in India.

## Methods

The present study utilized data from the fourth round of the National Family Health Survey (NFHS-4). NFHS-4 provides district level estimates of important indicators on population, health and nutrition for India and each State / Union territory. It provides data on 601,509 households, 699,686 women, and 103,525 men. The study utilizes data, which was collected from self-reported information pertaining to Tuberculosis in the household questionnaire. The specific question was, “Does any usual resident of your household suffer from tuberculosis?” the response to which helped in the detection of Tuberculosis (Additional file 1). Auxiliary information retrieved from a national report from the third round of National Family Health Survey (NFHS-3, 2005-06) is used for analysis.

### Sampling design and response rate

The NFHS-4 survey adopted a multistage stratified sampling design by considering the urban and rural areas as the natural strata. The 2011 census served as the sampling frame for the selection of Primary Sampling Units (PSUs). The PSUs in the survey were villages in rural areas. For urban areas, Census Enumeration Blocks (CEBs) were selected. Within each rural stratum, villages were selected from the sampling frame based on probability proportional to size (PPS). The final sample PSUs were selected through PPS sampling.

In urban areas, CEBs were also selected through PPS sampling. In every selected rural and urban PSU, selected PSUs with an estimated number of households were segmented. Two of the segments were randomly chosen for the survey using systematic sampling with probability proportional to segment size. Therefore, an NFHS-4 cluster is either a PSU or a segment of a PSU.

In the second stage, in every selected rural and urban cluster, households were randomly selected with systematic sampling. A total of 628,900 households were selected for the sample, of which 616,346 were occupied. Of the occupied households, 601,509 were successfully interviewed, a response rate of 98%. In the interviewed households, 723,875 eligible women age 15–49 years were identified for being interviewed individually of which 699,686 were interviewed, women, a response rate of 97%. Among males, there were 122,051 eligible men age 15–54 years in the households selected for the state module. Interviews were completed with 112,122 men, a response rate of 92%.

### Variables used

#### Response variables

Self-reported presence of Tuberculosis (TB) is the outcome variable.

#### Predictor variables

Based on the factors discussed in past studies, literature, exposure variables include various socio-economic, demographic and environmental indicators like age (< 15 years, 15-59 years and 60 and above), sex (male/female), educational attainment (no education, up to primary, up to secondary and higher), marital status (never married, currently married and widowed/ divorced/ separated/ disserted), place of residence (rural/ urban), wealth Index (poorest, poorer, middle, richer, richest), frequency of smoke inside house (never, less than daily and daily), type of fuel used for cooking (non- solid and solid), presence of separate room for cooking (yes/no), material of floor, wall and roof (natural, rudimentary and finished), number of people sleeping in a room (< 3 persons, 3 to 4, 5 to 6, 7 & above), shared toilet (no/yes) and potable water(yes/no).

### Statistical analysis

As the first step in the analysis, the study considered the prevalence of tuberculosis (TB) by background characteristics and then by household environment risk factors. This was followed by an estimation of the adjusted effects of the variables in the household environment, along with individual characteristics, on tuberculosis for the members in a household by using binary logistic regression model. The binary response (1 = presence of TB, 0 = absence of TB) for each respondent was related to a set of categorical predictors, X, by a *‘logit’* link function:

$$ \mathrm{logit}\ \left[\mathrm{P}\left(\mathrm{Y}=1\right)\right]=\mathrm{\ss}0+{\mathrm{\ss}}^{\ast}\mathrm{X}+\upepsilon $$

The parameter ***ß0*** estimates the log odds of TB for the reference group, while ***ß*** estimates the maximum likelihood, the differential log odds of TB associated with the set of predictors X, as compared to the reference group and ***ϵ*** represents the residuals in the model.

All the estimates and standard errors were adjusted for the multistage sampling design and clustering at the primary sampling unit and were weighted at the state level to give results that are unbiased and representative of the population. The statistical analysis was done in MS-Excel and STATA 13 software (Stata Corp, College Station, Texas).

## Results

### Prevalence of tuberculosis by background characteristics

It is seen that the prevalence of Tuberculosis (TB) varies according to different background characteristics, such as age, sex, educational attainment, marital status, place of residence and wealth index (Table 1). The overall prevalence of TB in India was 0.32%. It was found to be the highest among elderly people (0.9%). The prevalence of TB was also found to be higher in males (0.4%) than females (0.2%). Education has a significant association with TB. The prevalence of TB varied with educational attainment — people without education had the highest prevalence of TB (0.4%), while it was lowest among the people had higher education (0.04%). Those living in rural areas showed a higher prevalence of TB (0.34%) than those in urban places. As expected, people belonging to the most deprived (poorest wealth quintile) had the highest prevalence of TB (0.53%).

**Table 1:** Prevalence of Tuberculosis among household members by some selected background characteristics in India

Variables	Prevalence (%)	Number (N)
Age		
< 15 Years	0.06	847,380
15-59 years	0.33	1,737,560
60 & above	0.88	283,648
Sex		
Male	0.39	1,442,520
Female	0.22	1,426,523
Educational attainment		
No education	0.38	2,184,972
Up to primary	0.07	339,346
Up to secondary	0.08	325,790
Higher	0.04	18,688
Marital Status		
Never Married	0.16	596,279
Currently Married	0.45	1,373,339
Widowed/ Divorced/ Separated/ Disserted	0.68	167,581
Place of residence		
Urban	0.25	804,654
Rural	0.34	2,064,389
Wealth Index		
Poorest	0.53	609,790
Poorer	0.36	625,444
Middle	0.28	586,096
Richer	0.22	535,071
Richest	0.14	512,642
Overall	0.32	2,869,043

The physical environment within the household is closely associated with TB. Environmental risk factors, such as smoke inside the house, type of fuel used for cooking, separate kitchen, the material used for floors, roof and walls, number of persons sleeping in a room, sharing of toilets with other households, and potable water are strongly associated with TB as shown in Table 2. The prevalence of TB was found to be higher in households where there was regularly (daily) exposure to smoke within the house (0.4%). It is also seen that there is a strong association of the fuel used for cooking with Tuberculosis. Prevalence of TB was found to be higher in households using solid fuel for cooking (0.37%), and those that did not have a separate area for cooking (0.39%).

**Table 2:** Household environmental risk factors for Tuberculosis in India

Variables	Prevalence (%)	Number (N)
Smoke inside the house		
Never	0.26	1,462,050
Less than Daily	0.32	447,857
Daily	0.39	959,136
Fuel used for cooking		
Non-solid Fuel	0.21	1,023,975
Solid Fuel	0.37	1,601,004
Separate kitchen		
No	0.39	933,119
Yes	0.23	1,425,393
Material of floor		
Natural	0.42	1,139,694
Rudimentary	0.26	218,339
Finished	0.24	1,508,524
Material of wall		
Natural	0.41	568,060
Rudimentary	0.42	306,853
Finished	0.27	1,985,085
Material of roof		
Natural	0.51	206,653
Rudimentary	0.39	142,116
Finished	0.29	2,422,173
Number of persons sleeping per room		
< 3 persons	0.33	2,124,315
3 to 4	0.23	655,126
5 to 6	0.12	73,421
7 & above	0.18	15,766
Share toilet with other household		
No	0.23	1,508,130
Yes	0.34	246,380
Potability of water		
No	0.35	1,725,274
Yes	0.23	1,143,306
Overall	0.32	2,868,580

Predictably, persons living in poor quality dwellings have a higher risk of Tuberculosis. The prevalence of TB was higher — respectively 0.42, 0.41, and 0.51% —in households with mud (or other naturally available material) floors, walls, and roofs. TB was considerably higher (0.34%) in households that share toilets with other households, and those not using potable water (0.4%).

**Fig. 1:**
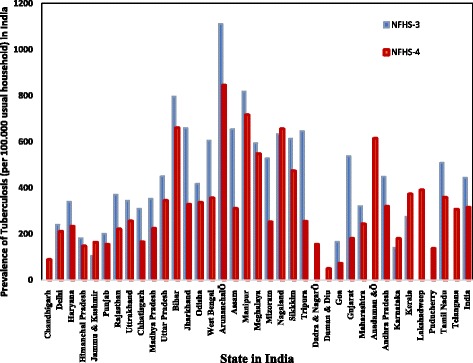
Number of persons per 100,000 usual household residents suffering from any tuberculosis by state, India (2005-06, 2015-16)

Comparison of state wise prevalence (number of persons suffering from TB per 100,000 usual residents of households) is shown in Fig. 1. Data from two rounds of National Family Health Survey (NFHS-3 and NFHS-4) was used. It is seen that there is a significant increase in the prevalence of TB in the states of Jammu & Kashmir, Nagaland, Karnataka, and Kerala. In the other states, a sharp decline is seen.

Table 3 presents the result of logistic regression, which helps in the understanding of the factors associated with the prevalence of TB in the Indian population. Two models were used to assess the factors: Model-1 is concerned with socio-economic variables (including age, sex, educational attainment, marital status, place of residence, wealth index), while Model-2 considers variables related to the household environment (smoke inside house, fuel used for cooking, separate kitchen, the material of floor, material of wall, the material of roof, a room used for sleeping, sharing of toilets with other households and use of potability of water) in addition to those considered in Model-1.

**Table 3:** Adjusted effect of household environment and other selected variables on tuberculosis among member of household in India

Variables	Model-1		Model-2	
	Odds Ratio	95% C.I.	Odds Ratio	95% C.I.
Age				
< 15 Years®				
15-59 years	1.58^*^	1.27- 1.97	1.39	1.00-1.91
60 & above	3.06^*^	2.44-3.84	2.94^*^	2.11-4.09
Sex				
Male®				
Female	0.51^*^	0.48-0.53	0.58^*^	0.54-0.62
Educational attainment				
No education®				
Up to primary	1.05	0.68-1.63	1.25	0.66-2.38
Up to secondary	0.51^*^	0.44-0.60	0.61^*^	0.49-0.76
Higher	0.40^*^	0.24-0.68		
Marital Status				
Never Married®				
Currently Married	1.87^*^	1.74-2.02	1.58^*^	1.41-1.76
Widowed/ Divorced/ Separated/ Disserted	2.29^*^	2.07-2.53	1.80^*^	1.54-2.10
Place of residence				
Urban®				
Rural	0.82^*^	0.77-0.87	0.86^*^	0.79-0.93
Wealth Index				
Poorest®				
Poorer	0.70^*^	0.67-0.74	0.68^*^	0.59-0.78
Middle	0.54^*^	0.51-0.57	0.55^*^	0.47-0.64
Richer	0.40^*^	0.37-0.43	0.43^*^	0.36-0.52
Richest	0.22^*^	0.20-0.24	0.26^*^	0.21-0.32
Smoke inside the house				
Never®				
Less than Daily			1.38^*^	1.25-1.52
Daily			1.49^*^	1.39-1.61
Fuel used for cooking				
Non-solid Fuel®				
Solid Fuel			1.03	0.94-1.14
Separate kitchen				
No®				
Yes			0.95	0.88-1.03
Material of floor				
Natural®				
Rudimentary			1.27^*^	1.13-1.43
Finished			1.12^*^	1.01-1.25
Material of wall				
Natural®				
Rudimentary			1.07	0.95-1.20
Finished			0.74^*^	0.66-0.83
Material of roof				
Natural®				
Rudimentary			0.82	0.66-1.02
Finished			0.90	0.79-1.04
Room used for sleeping				
< 3 person®				
3 to 4			0.95	0.88-1.03
5 to 6			0.87	0.70-1.09
7 & above			0.83	0.49-1.41
Share toilet with other household				
No®				
Yes			1.23^*^	1.13-1.36
Potability of water				
No®				
Yes			1.1^*^	1.03-1.18

*Note*: ® Reference category, *P < 0.05

The adjusted effects, as estimated from Model-1, show that age, sex, educational attainment, marital status, place of residence, wealth index have a statistically significant association with Tuberculosis. Older people in the age group of 16–59 years (OR = 1.6; CI = 1.3-2.0) and those aged 60 years and above (OR = 3.1; CI = 2.4-3.8) are more prone to getting Tuberculosis as compared to individuals age less than 15 years. Females are less likely (OR = 0.51; CI = 0.5-0.6) to get Tuberculosis than males. It is also seen that educational attainment has a strong association with TB. Persons who had studied up to the secondary (OR = 0.5; CI = 0.4-0.6) level and higher (OR = 0.4; CI = 0.2-0.7) are seen to be at lower risk of getting tuberculosis as compared with those counterparts who had no education. Further, people living in urban areas are more prone to the infection then rural people. As anticipated, poor people are 78% more likely to get TB than the people belonging to the richest wealth quintile in India.

The variables associated with the household environment, which are considered in Model-2, also show statistically significant association with Tuberculosis. Persons who are regularly (daily) exposed to second-hand smoke inside their homes are more prone to getting tuberculosis (OR = 1.49; CI = 1.39-1.61) as compared those who are not. Households that use solid fuel for cooking and do not have a separate kitchen are more prone to infection with tuberculosis than those using non-solid fuel for cooking and having a separate room for cooking. The results are not statistically significant. Further, households having a finished wall (OR = 0.7; CI = 0.6-0.8) are less likely to get TB than those with mud walls. The predictor variables such as roof material and room for sleeping also have an association with TB; however, the results are not statistically significant. Households that shared their toilet with other households are more likely to get Tuberculosis (OR = 1.2; CI = 1.1-1.4) as compared to those that do not. Further, households that are not using potable water (or are not treating water to make it safer for consumption) are ten times more likely to get Tuberculosis than the households that use potable water.

## Discussion

The World Health Organization has raised many important issues during discussions in various International fora. Among these, public health, environmental and social determinants of health are the most crucial. WHO (2017) has estimated that about 12.6 million deaths each year are attributable to morbid environments — almost one-fourth of total global deaths [18, 19]. Environmental risk factors, such as air, water and soil pollution, chemical exposures, climate change and ultraviolet radiation, contribute to more than 100 diseases and injuries. The objective of present study was to examine the association between household living environment and prevalence of Tuberculosis in India. The results demonstrate the effects of household environmental factors on Tuberculosis (TB), which suggests that the likelihood of the presence of self-reported TB is higher for elderly respondent i.e., those above 60 years of age. The elderly are 3.1 times more prone to tuberculosis. This finding is found to be consistent with the findings of other reported studies, suggesting that are difficulties in diagnosing the presence of TB in the elderly because only a small proportion of them suffer from respiratory disorders. Additionally, factors like tobacco use, low socio-economic status, previous disease, longer delays in seeking medical attention and very high rates of adverse reactions during treatment exacerbates the likelihood of TB in the Indian elderly [20]. Elderly suffer from multiple morbidities along with TB face a higher burden of TB, in terms of both health and monetary hardships. The 2010 report of Global Burden of Disease estimates that, globally, 57% of all tuberculosis deaths occur among people aged 50 years or above, with more than half of these deaths in those aged more than 65 years of age. Estimates from studies suggest that around 51% of TB Disability-Adjusted Life Years (DALYs) are contributed by patients aged 50 years and older [21]. The major contributing factor is the increased level of susceptibility to infectious diseases and weakened immune system amongst the elderly [22–24]. Therefore, this group requires special attention which should include early screening and initiation of treatment, as well as proper nutritional supervision.

The results also show that the prevalence of TB was higher among males than females. Evidence from previous studies are also consistent with this finding. One of the major reasons could be that Indian men are more exposed to smoking and drinking [3]. Further, men are expected to be in greater contact with people who suffer from active Tuberculosis (TB) than women. While the World Health Organization (WHO) stated that the global male to female prevalence ratio of tuberculosis (TB) is in favour of males, which further widens with increasing age [25, 26]. However, today, TB is the leading cause of death for women globally [27]. The Sustainable Development Goals (SDGs) have stressed on gender equality and health, supported by specific targets to eliminate make available gender-specific data on TB incidence (target 3.3). TB has social stigmas attached to it which affects men and women differently. Studies in India suggest that women suffering from TB are ostracized and ill-treated. The consequences include social isolation, reduced chances of marriage, rejection, and harassment by in-laws and spouse. In the case of men, the likely consequences are social isolation, loss of income and economic hardships [9, 27, 28].

Place of residence also has a significant association with Tuberculosis. People living in urban areas are more prone infection than those in rural areas. Extant literature shows rural areas as pockets of TB which is further accelerated by dynamics of knowledge about the disease. These areas are marked by stigmatization, resulting in underutilization of treatment services, delay in diagnosis, and poor treatment fulfillment [29, 30]. Prevalence of TB is also seen to vary with educational attainment. People without education had the highest prevalence of TB. Educational attainment has a strong association with TB. Respondents who had studied up to the secondary level and higher are at a lower risk of getting Tuberculosis as compared to those who had no education.

The likelihood of TB was found to be higher (0.34%) among respondents whose households shared toilet facilities with others, and households with mud walls. TB was considerably higher in households where they usually share their toilet with other households. It was also higher (0.40%, and 1.2 times more likely) in households that did not use potable water. It was calculated that households that shared their toilet with other households are 1.2 times more likely to be infected with Tuberculosis as compared to the households that do not share.

Predictor variables such as the material used in roof and room used for sleeping also have an association with TB. Predictably, it was found that those living in the poor-quality housing (where the material of wall, roof, and the floor is made of mud or other natural material) are at higher risk of Tuberculosis. Prevalence of TB was higher — 0.42, 0.41 and 0.51% respectively in households where the floors, roofs, and walls were made of the mud or natural material. Households with finished walls are 0.7 times as less likely to be infected with TB than households with mud walls. Evidence from the Indian study which shows that ORs for poor-quality housing, toilet facilities and water supply were significant. In multivariate logistic regression analysis, the age, level of education, household crowding, type of housing and water supply in the household was found to be independently and significantly associated with a higher risk of TB [26].

Recent estimates in the World Bank’s profile of India’s poverty pointed out that one out of five Indian belong to the below poverty line (deprived section of society), or BPL group [31]. As expected, people belonging to the most deprived (the poorest wealth quintile) show the highest prevalence of TB (0.53%). As anticipated, poor people are 78% more likely to get TB than the people in India’s richest wealth quintile. Owing to the living and working conditions, it is the impoverished class which is more vulnerable to the disease. Moreover, TB often results in a vicious circle of poverty and illness. Estimates suggest that on average, 20 − 30% of annual household income is lost due to TB [27, 32]. Studies also suggest that newly-diagnosed elderly TB patients are more likely to be lost to follow-up, die, or report drug side effects [33].

Earlier research suggests that the likelihood of TB is higher among respondents who were exposed daily to smoke in their homes. This study found that prevalence of TB was higher (0.40%) in households where there is daily exposure to smoke (0.4%). Households in which the family members are exposed to smoke (second-hand smoke) daily are 1.5 times more prone to getting tuberculosis as compared with households where people do not smoke inside the house. Smoke exposure may be caused by tobacco (from smoking or second-hand smoke), or from the burning of biomass. Similar inferences are also drawn from the present study [9]. Past studies strongly suggest that exposure to cooking smoke considerably increases the risk of tuberculosis in India [3, 10, 34, 35]. Often, women are more exposed to smoke inside household which puts them at greater risk of TB [36].

The risk of tuberculosis is influenced by factors, such as age, sex, nutrition levels (biological factors); tobacco smoking, alcoholism (behavioral factors); poverty, overcrowding and poor housing (socio-economic factors). The environment in which one lives has a major influence on the epidemiology of TB. Factors like exposure to smoke inside the house, exposure to second-hand smoke, number of people in each room (i.e., the crowing index), the material of construction of the house, sanitation facilities affect TB status [3, 37, 38]. There is a higher risk of TB if the households share their toilets and kitchens with people suffering from TB [3, 39]. The study also found the association of cooking fuel and Tuberculosis. Prevalence of TB was found to be higher in households that normally use solid fuel for cooking (0.37%), and those that did not have a separate kitchen for cooking (0.39%). Households using solid fuel for cooking and not having separate kitchen are more prone to TB infection in India than households using non-solid fuel for cooking and having a separate room for a kitchen space. There is also strong evidence that exposure to cooking smoke considerably increases the risk of tuberculosis in India. Because women are more exposed to kitchen smoke, they are more vulnerable to TB [3, 10, 34–36].

The use of a cross-sectional survey to collect data may have underestimated the true prevalence of Tuberculosis. The results of self-reported morbidities could be biased due to subjectivity in responses. Recall bias may also have affected the estimated prevalence of Tuberculosis.

## Conclusion

However, the results of the analysis in this study provide compelling evidence that a contaminated household environment increases the risk of Tuberculosis in India. This is why a clean environment must be promoted to eliminate TB, still a significant cause of deaths in India. The study found environmental risk factors for TB, such as smoke inside the house, type of fuel used for cooking, separate kitchen space, floor, wall and roof materials, the number of persons sleeping in a room, sharing of toilets with other households, and non-use of potable water. Besides the environmental factors, individual characteristics like age, sex, educational attainment, marital status, place of residence and wealth index are also strongly associated with TB.

## Additional file

Additional file 1: Household Questionnaire of NFHS-4 Survey. (PDF 175 kb)

## Abbreviations

CEBs: Census Enumeration Blocks
DALYs: Disability-Adjusted Life Years
NFHS: National Family and Health Survey
PSUs: Primary Sampling Units
SDGs: Sustainable Development Goals
TB: Tuberculosis
WHO: World Health Organization
