# Supplementary material for: Potential effect of household environment on prevalence of tuberculosis in India: evidence from the recent round of a cross-sectional survey
Source: BMC Pulm Med. 2018 May 3;18:66. doi: 10.1186/s12890-018-0627-3 (PMC5934826; doi:10.1186/s12890-018-0627-3)
Supplement: Supplementary file 1 — Household Questionnaire of NFHS-4 Survey. (PDF 175 kb) [file 12890_2018_627_MOESM1_ESM.pdf]

राष्ट्रीय परिवार स्वास्थ्य सर्वेक्षण, भारत 2015-16 (NFHS-4)
परिवार प्रश्नावली [STATE NAME]

NATIONAL FAMILY HEALTH SURVEY, INDIA 2015-2016 (NFHS-4)
HOUSEHOLD QUESTIONNAIRE [STATE NAME]

CONFIDENTIAL
For research purposes only

IDENTIFICATION

STATE ______________________

DISTRICT ______________________

TEHSIL/TALUK ______________________

CITY/TOWN/VILLAGE ______________________

TYPE OF PSU (URBAN = 1, RURAL = 2) . . . .

PSU NUMBER . . . .

STRUCTURE NUMBER . . . .

HOUSEHOLD NUMBER . . . .

NAME OF HOUSEHOLD HEAD ______________________

ADDRESS OF HOUSEHOLD ______________________

IS HOUSEHOLD SELECTED FOR THE STATE MODULE? (YES = 1, NO = 2) . . . .

INTERVIEWER VISITS

	1	2	3	FINAL VISIT
DATE	______	______	______	DAY<br>MONTH<br>YEAR
INTERVIEWER'S NAME	______	______	______	INT. NO.
RESULT CODE*	______	______	______	RESULT CODE*
NEXT VISIT: DATE<br>TIME	______<br>______	______<br>______		TOTAL NUMBER OF VISITS
SUPERVISOR'S NAME	______			SUPERVISOR NUMBER

*RESULT CODES:
1 COMPLETED
2 NO HOUSEHOLD MEMBER AT HOME OR NO COMPETENT RESPONDENT AT HOME AT TIME OF VISIT
3 ENTIRE HOUSEHOLD ABSENT FOR EXTENDED PERIOD OF TIME
4 POSTPONED
5 REFUSED
6 DWELLING VACANT OR ADDRESS NOT A DWELLING
7 DWELLING DESTROYED
8 DWELLING NOT FOUND
9 OTHER ______________________ (SPECIFY)

TOTAL PERSONS IN HOUSEHOLD

TOTAL ELIGIBLE WOMEN

TOTAL ELIGIBLE MEN

LINE NO. OF RESPONDENT TO HOUSEHOLD QUESTIONNAIRE

**LANGUAGE CODES:

01 ASSAMESE	08 MALAYALAM	15 TAMIL
02 BENGALI	09 MANIPURI	16 TELUGU
03 GUJARATI	10 MARATHI	17 URDU
04 HINDI	11 NEPALI	18 ENGLISH
05 KANNADA	12 ORIYA	19 GARO
06 KASHMIRI	13 PUNJABI	20 KHASI
07 KONKANI	14 SINDHI	96 OTHER ______ SPECIFY

LANGUAGE OF QUESTIONNAIRE **HINDI 0 4

**RESPONDENT'S MOTHER TONGUE ______________________

**LANGUAGE OF INTERVIEW ______________________

TRANSLATOR USED? (YES = 1, NO = 2) . . . .

INTRODUCTION AND INFORMED CONSENT

नमस्ते। मेरा नाम _________ है। मैं (NAME OF ORGANIZATION) के साथ काम कर रहा/ रही हूँ। हम पूरे भारत में स्वास्थ्य पर एक सर्वेक्षण कर रहे हैं। जो जानकारी हम परिवार कल्याण और स्वास्थ्य के बारे में घरों और व्यक्तियों से इकठ्ठी करेंगे वो सरकार को स्वास्थ्य सेवाएं बनाने में मदद करेगी। आपका परिवार इस सर्वेक्षण के लिए चुना गया है। मैं आपसे आपके परिवार के बारे में कुछ सवाल पूछना चाहूँगा/ चाहूँगी। इन सवालों में लगभग 25-35 मिनट लगेंगे। आपके सारे जवाब गुप्त रखे जायेंगे और हमारे सर्वेक्षण के सदस्यों के अलावा किसी को भी नहीं बताये जायेंगे। आपका इस सर्वेक्षण में भाग लेना स्वैच्छिक हैं। अगर आप मेरे किसी सवाल का जवाब नहीं देना चाहते, तो मुझे बता दीजिये और मैं अगले सवाल पर चला जाऊँगा/ जाऊँगी या आप किसी भी समय यह बातचीत रोक सकते हैं।

क्या आप मुझसे कुछ सवाल पूछना चाहती / चाहते है?
ANSWER ANY QUESTIONS AND ADDRESS RESPONDENT'S CONCERNS.

यदि आपको इस सर्वेक्षण के बारे में और जानकारी चाहिए तो आप इस कार्ड पर दिए गए नाम वाले व्यक्ति को संपर्क करें।
GIVE CARD WITH CONTACT INFORMATION.

क्या आप इस सर्वेक्षण में भाग लेने के लिए सहमत हैं?

Namaste. My name is _______. I am working with (NAME OF ORGANIZATION). We are conducting a survey about health all over India. The information on family welfare and health that we collect from households and individuals will help the government to plan health services. Your household was selected for the survey. I would like to ask you some questions about your household. The questions usually take about 25-35 minutes. All of the answers you give will be confidential and will not be shared with anyone other than members of our survey team. Your participation in the survey is voluntary. If I ask you any question you don't want to answer, just let me know and I will go on to the next question or you can stop the interview at any time.

If you have any questions about this survey you may ask me.
ANSWER ANY QUESTIONS AND ADDRESS RESPONDENT'S CONCERNS.

If you have any further questions about this survey you may contact the persons listed on this card.
GIVE CARD WITH CONTACT INFORMATION.

Do you agree to participate in this survey?

SIGNATURE OF INTERVIEWER ______________________ DATE ______________

RESPONDENT AGREES TO BE INTERVIEWED . . . 1 → BEGIN INTERVIEW

RESPONDENT DOES NOT AGREE TO BE INTERVIEWED . . . 2 → END

THIS PAGE IS INTENTIONALLY BLANK

HOUSEHOLD SCHEDULE

अब हम उन लोगों के बारे में कुछ जानकारी चाहेंगे जो सामान्यत: आप के घर में रहते हैं या जो अभी आपके साथ रह रहे हैं।
Now we would like some information about the people who usually live in your household or who are staying with you now.

LINE NO.	USUAL RESIDENTS AND VISITORS	RELATIONSHIP TO HEAD OF HOUSEHOLD	SEX	RESIDENCE		AGE	MARITAL STATUS	ELIGIBILITY			BIRTH REGIS-TRATION
	कृपया मुझे उन व्यक्तियों के नाम बतायें जो सामान्यत: आपके घर में रहते हैं और वे अतिथि जो पिछली रात इसी घर में ठहरे थे। शुरुआत घर के मुखिया से करें।<br>Please give me the names of the persons who usually live in your household and guests of the household who stayed here last night, starting with the head of the household.<br>AFTER LISTING THE NAME, RELATIONSHIP, SEX, RESIDENCE, AND AGE FOR EACH PERSON; ASK QUESTIONS 7A(a-c) TO BE SURE THAT THE LISTING IS COMPLETE.	घर के मुखिया से (NAME) का रिश्ता क्या है?<br>What is the relationship of (NAME) to the head of the household?<br>(A)	क्या (NAME) पुरुष है या स्त्री?<br>Is (NAME) male or female?	क्या (NAME) सामान्यत: यहीं (रहते/रहती) हैं?<br>Does (NAME) usually live here?	क्या (NAME) पिछली रात यहीं (ठहरे थे/ठहरी थीं)?<br>Did (NAME) stay here last night?	(NAME) की आयु क्या है?<br>How old is (NAME)?<br>RECORD COM-PLETED YEARS.<br>(B)	**IF AGE 13 OR OLDER**<br>(NAME) की वर्तमान वैवाहिक स्थिति क्या है?<br>What is the current marital status of (NAME)?<br>(C)	CIRCLE LINE NUMBER OF ALL WOMEN AGE 15-49	**IF HOUSE-HOLD IS SELEC-TED FOR STATE MODULE**<br>CIRCLE LINE NUMBER OF ALL MEN AGE 15-54	CIRCLE LINE NUMBER OF ALL CHILDREN AGE 0-5	**IF AGE 0-4**<br>क्या (NAME) के जन्म का प्रमाणपत्र है? IF NO: क्या (NAME) के जन्म का कभी नागरिक प्राधिकरण में पंजीकरण किया गया है?<br>Does (NAME) have a birth certificate? IF NO: Has (NAME)'s birth ever been regis-tered with the civil authority?<br>(D)
(1)	(2)	(3)	(4)	(5)	(6)	(7)	(8)	(9)	(10)	(11)	(12)
01			M F<br>1 2	YES NO<br>1 2	YES NO<br>1 2	IN YEARS		01	01	01	C R N DK<br>1 2 3 8
02			1 2	1 2	1 2			02	02	02	1 2 3 8
03			1 2	1 2	1 2			03	03	03	1 2 3 8
04			1 2	1 2	1 2			04	04	04	1 2 3 8
05			1 2	1 2	1 2			05	05	05	1 2 3 8
06			1 2	1 2	1 2			06	06	06	1 2 3 8
07			1 2	1 2	1 2			07	07	07	1 2 3 8
08			1 2	1 2	1 2			08	08	08	1 2 3 8
09			1 2	1 2	1 2			09	09	09	1 2 3 8
10			1 2	1 2	1 2			10	10	10	1 2 3 8
11			1 2	1 2	1 2			11	11	11	1 2 3 8

LINE NO.	SURVIVORSHIP AND RESIDENCE OF BIOLOGICAL PARENTS				EDUCATION					AADHAAR CARD
	**IF AGE 0-17**				**IF AGE 5 OR OLDER**		**IF AGE 5-18**			
	क्या (NAME) को जन्म देनेवाली माता जीवित है?<br>Is (NAME)'s natural mother alive?	क्या (NAME) को जन्म देने वाली माँ सामान्यतः इस घर में रहती हैं या वे कल रात यहाँ मेहमान थी?<br>IF YES: उनका नाम क्या है?<br>Does (NAME)'s natural mother usually live in this household or was she a guest last night?<br>IF YES: What is her name?<br>IF YES: RECORD MOTHER'S LINE NO. IF NO: RECORD '00'.	क्या (NAME) के सगा पिता जीवित है?<br>Is (NAME)'s natural father alive?	क्या (NAME) का सगा पिता इस घर में रहते है या वे कल रात यहाँ मेहमान थे?<br>IF YES: उनका नाम क्या है?<br>Does (NAME)'s natural father usually live in this household or was he a guest last night?<br>IF YES: What is his name?<br>IF YES: RECORD FATHER'S LINE NO. IF NO: RECORD '00'.	क्या (NAME) कभी स्कूल (गया/गयी) हैं?<br>Has (NAME) ever attended school?	(NAME) ने कौन-सा उच्चतम दर्जा पास किया है?<br>What is the highest standard (NAME) has completed?<br>(E)	क्या (NAME) स्कूल वर्ष 2014-2015 के दौरान कभी स्कूल या कॉलेज गया/गयी हैं?<br>Did (NAME) attend school or college at any time during the 2014-15 school year?	(इस/उस) स्कूल वर्ष के दौरान (NAME) किस दर्जे/वर्ष में जा (रहा है/था) (रही है/थी)?<br>During (this/that) school year, what standard/ year (is/was) (NAME) attending?<br>(E)	**IF NO ON Q.19**<br>(NAME) के स्कूल न जाने का मुख्य कारण क्या है?<br>What is the main reason (NAME) is not attending school?<br>(F)	क्या (NAME) के पास आधार कार्ड है?<br>Does (NAME) have an Aadhaar card?
	(13)	(14)	(15)	(16)	(17)	(18)	(19)	(20)	(21)	(21A)
01	Y N DK<br>1 2 8<br>GO TO 15	LINE NO.	Y N DK<br>1 2 8<br>GO TO 17	LINE NO.	YES NO<br>1 2<br>GO TO 21A	STANDARD	YES NO<br>1 2<br>GO TO 21	STANDARD<br>GO TO 21A	REASON	YES NO<br>1 2
02	1 2 8<br>GO TO 15		1 2 8<br>GO TO 17		1 2<br>GO TO 21A		1 2<br>GO TO 21	GO TO 21A		YES NO<br>1 2
03	1 2 8<br>GO TO 15		1 2 8<br>GO TO 17		1 2<br>GO TO 21A		1 2<br>GO TO 21	GO TO 21A		YES NO<br>1 2
04	1 2 8<br>GO TO 15		1 2 8<br>GO TO 17		1 2<br>GO TO 21A		1 2<br>GO TO 21	GO TO 21A		YES NO<br>1 2
05	1 2 8<br>GO TO 15		1 2 8<br>GO TO 17		1 2<br>GO TO 21A		1 2<br>GO TO 21	GO TO 21A		YES NO<br>1 2
06	1 2 8<br>GO TO 15		1 2 8<br>GO TO 17		1 2<br>GO TO 21A		1 2<br>GO TO 21	GO TO 21A		YES NO<br>1 2
07	1 2 8<br>GO TO 15		1 2 8<br>GO TO 17		1 2<br>GO TO 21A		1 2<br>GO TO 21	GO TO 21A		YES NO<br>1 2
08	1 2 8<br>GO TO 15		1 2 8<br>GO TO 17		1 2<br>GO TO 21A		1 2<br>GO TO 21	GO TO 21A		YES NO<br>1 2
09	1 2 8<br>GO TO 15		1 2 8<br>GO TO 17		1 2<br>GO TO 21A		1 2<br>GO TO 21	GO TO 21A		YES NO<br>1 2
10	1 2 8<br>GO TO 15		1 2 8<br>GO TO 17		1 2<br>GO TO 21A		1 2<br>GO TO 21	GO TO 21A		YES NO<br>1 2
11	1 2 8<br>GO TO 15		1 2 8<br>GO TO 17		1 2<br>GO TO 21A		1 2<br>GO TO 21	GO TO 21A		YES NO<br>1 2

TICK HERE IF CONTINUATION QUESTIONNAIRE USED ☐

7A यह सुनिश्चित करने के लिये की मैंने आपके पूरे परिवार का सूचीकरण कर लिया है:
Just to make sure that I have a complete household listing:

a) क्या यहां कोई अन्य व्यक्ति हैं जिन को हमने इस सूची में शामिल नहीं किया है जैसे कि छोटे बच्चे या शिशु?
Are there any other persons such as small children or infants that we have not listed?
YES → ENTER EACH IN TABLE NO ☐

b) क्या यहां ऐसे कोई अन्य लोग सामान्यत: रहते हैं जो आपके परिवार के सदस्य नही है जैसे घरेलू नौकर या दोस्त?
Are there any other people who may not be members of your family such as domestic servants, lodgers or friends who usually live here?
YES → ENTER EACH IN TABLE NO ☐

c) क्या पिछली रात यहां कोई मेहमान, अस्थायी आगन्तुक अथवा कोई अन्य व्यक्ति ठहरे थे जो इस सूची में शामिल नहीं है?
Are there any guests or temporary visitors staying here, or anyone else who stayed here last night, who have not been listed?
YES → ENTER EACH IN TABLE NO ☐

(A) CODES FOR Q. 3

RELATIONSHIP TO HEAD OF HOUSEHOLD:

01 = HEAD
02 = WIFE OR HUSBAND
03 = SON OR DAUGHTER
04 = SON-IN-LAW OR DAUGHTER-IN-LAW
05 = GRANDCHILD
06 = PARENT
07 = PARENT-IN-LAW
08 = BROTHER OR SISTER
09 = BROTHER-IN-LAW OR SISTER-IN-LAW
10 = NIECE/NEPHEW
11 = OTHER RELATIVE
12 = ADOPTED/FOSTER/STEP-CHILD
13 = DOMESTIC SERVANT
14 = OTHER NOT RELATED
98 = DON'T KNOW

(B) CODES FOR Q. 7

AGE:

00 = AGE LESS THAN ONE YEAR
95 = AGE 95 YEARS OR MORE

(C) CODES FOR Q. 8

MARITAL STATUS:

1 = CURRENTLY MARRIED
2 = MARRIED, BUT GAUNA NOT PERFORMED
3 = WIDOWED
4 = DIVORCED
5 = SEPARATED
6 = DESERTED
7 = NEVER MARRIED
8 = DON'T KNOW

(D) CODES FOR Q. 12

BIRTH REGISTRATION:

1 = C = CERTIFICATE
2 = R = REGISTRATION
3 = N = NEITHER
8 = DK = DON'T KNOW

(E) CODES FOR Q.18 AND Q.20

EDUCATION STANDARD:

00 = LESS THAN 1 YEAR COMPLETED OR PRE-PRIMARY
98 = DON'T KNOW

(F) CODES FOR 21

REASON FOR NOT ATTENDING SCHOOL:

01 = SCHOOL TOO FAR AWAY
02 = TRANSPORT NOT AVAILABLE
03 = FURTHER EDUCATION NOT CONSIDERED NECESSARY
04 = REQUIRED FOR HOUSEHOLD WORK
05 = REQUIRED FOR WORK ON FARM/FAMILY BUSINESS
06 = REQUIRED FOR OUTSIDE WORK FOR PAYMENT IN CASH OR KIND
07 = COSTS TOO MUCH
08 = NO PROPER SCHOOL FACILITIES FOR GIRLS
09 = NOT SAFE TO SEND GIRLS
10 = NO FEMALE TEACHER
11 = REQUIRED FOR CARE OF SIBLINGS
12 = NOT INTERESTED IN STUDIES
13 = REPEATED FAILURES
14 = GOT MARRIED
15 = DID NOT GET ADMISSION
96 = OTHER
98 = DON'T KNOW

NO.	QUESTIONS AND FILTERS	CODING CATEGORIES	SKIP
22	कोई व्यक्ति प्रायः कितनी बार आपके घर के अंदर धुम्रपान करता है, क्या आप कहेंगे रोजाना, हप्ते में एकबार, महिने में एक बार, कभी नहीं?<br><br>How often does anyone smoke inside your house? Would you say daily, weekly, monthly, less than monthly, or never?	DAILY . . . . 1<br>WEEKLY . . . . 2<br>MONTHLY . . . . 3<br>LESS THAN MONTHLY . . . . . 4<br>NEVER 5	
23	क्या आपके घर का कोई सामान्य निवासी तपेदिक [टी बी] रोग से पीड़ित है?<br><br>Does any usual resident of your household suffer from tuberculosis?	YES . . . . 1<br>NO 2	 → 25
24	तपेदिक [टी बी] रोग से कौन पीड़ित है?<br>कोई अन्य?<br><br>Who suffers from tuberculosis?<br>Anyone else?<br><br>RECORD LINE NUMBER(S).<br>IF NO MORE TB CASES, RECORD '95'.	24A FOR EACH PERSON, ASK:<br>क्या (NAME) ने तपेदिक [टी बी] के लिए चिकित्सकीय इलाज कराया है?<br>IF YES, ASK: (NAME) कहाँ गयें?<br><br>Has (NAME) received medical treatment for the tuberculosis?<br>IF YES, ASK: Where did (NAME) go?	

		YES, PUBLIC ONLY	YES, PRIVATE ONLY	YES, BOTH	NO
LINE NO.	☐☐	1	2	3	4
LINE NO.	☐☐	1	2	3	4
LINE NO.	☐☐	1	2	3	4
LINE NO.	☐☐	1	2	3	4

NO.	QUESTIONS AND FILTERS	CODING CATEGORIES	SKIP
25	आपके घर के सदस्यों के लिए पीने के पानी का मुख्य स्रोत क्या है?<br><br>What is the main source of drinking water for members of your household?	**PIPED WATER**<br>PIPED INTO DWELLING . . . . . 11<br>PIPED TO YARD/PLOT . . . . . 12<br>PUBLIC TAP/STANDPIPE . . . . . 13<br>TUBE WELL OR BOREHOLE . . . . 21<br>**DUG WELL**<br>PROTECTED WELL . . . . . . 31<br>UNPROTECTED WELL . . . . . . 32<br>**WATER FROM SPRING**<br>PROTECTED SPRING . . . . . . 41<br>UNPROTECTED SPRING . . . . . 42<br>RAINWATER . . . . 51<br>TANKER TRUCK . . . . 61<br>CART WITH SMALL TANK . . . . . 71<br>SURFACE WATER (RIVER/DAM/ LAKE/POND/STREAM/CANAL/ IRRIGATION CHANNEL) . . . . . 81<br>BOTTLED WATER . . . . 91<br>COMMUNITY RO PLANT . . . . . 92<br><br>OTHER ________ 96<br>(SPECIFY)	11, 12 → 29<br><br>51 → 29
26	पानी का स्रोत कहां पर है?<br><br>Where is the water source located?	IN OWN DWELLING . . . . 1<br>IN OWN YARD/PLOT . . . . . . 2<br>ELSEWHERE 3	1, 2 → 29

NO.	QUESTIONS AND FILTERS	CODING CATEGORIES	SKIP
27	वहां एक बार जाने में, पानी लेने में, और वापस आने में कितना समय लगता है?<br><br>How long does it take to go there, get water, and come back in one trip?	MINUTES . . . . [ ][ ][ ]<br><br>DELIVERED TO DWELLING . . . . 000<br>ON THE PREMISES . . . . 996<br>DON'T KNOW 998	000, 996 → 29
28	आपके घर के लिए इस स्रोत से पानी लाने के लिए सामान्यतः कौन जाता है?<br><br>Who usually goes to this source to fetch the water for your household?	ADULT WOMAN . . . . 1<br>ADULT MAN . . . . 2<br>FEMALE CHILD<br>UNDER AGE 15 YEARS . . . . 3<br>MALE CHILD<br>UNDER AGE 15 YEARS . . . . 4<br>OTHER ________________ 6<br>(SPECIFY)	
29	क्या आपके घर के सदस्य पीने के पानी को सुरक्षित बनाने के लिए कुछ करते हैं?<br>Does this household do anything to the water to make it safer to drink?	YES . . . . 1<br>NO . . . . 2<br>DON'T KNOW 8	2, 8 → 31
30	पीने के पानी को सुरक्षित बनाने के लिए सामान्यतः आपका परिवार क्या करता है? कोई अन्य?<br><br>What does this household usually do to make the water safer to drink?<br>Anything else?<br><br>RECORD ALL MENTIONED.	BOIL . . . . A<br>USE ALUM . . . . B<br>ADD BLEACH/CHLORINE TABLETS . C<br>STRAIN THROUGH A CLOTH . . . . D<br>USE WATER FILTER (CERAMIC/<br>SAND/COMPOSITE/ETC.) . . . . E<br>USE ELECTRONIC PURIFIER . . . . F<br>LET IT STAND AND SETTLE . . . . G<br>OTHER ________________ X<br>(SPECIFY)<br>DON'T KNOW . . . . Z	
31	आपके परिवार के सदस्य सामान्यतः किस प्रकार की शौच सुविधा का इस्तेमाल करते हैं?<br><br>What kind of toilet facility do members of your household usually use?	**FLUSH OR POUR FLUSH TOILET**<br>FLUSH TO PIPED SEWER<br>SYSTEM . . . . 11<br>FLUSH TO SEPTIC TANK . . . . 12<br>FLUSH TO PIT LATRINE . . . . 13<br>FLUSH TO SOMEWHERE ELSE . . . . 14<br>FLUSH, DON'T KNOW WHERE . . . . 15<br>**PIT LATRINE**<br>VENTILATED IMPROVED<br>PIT (VIP)/BIOGAS LATRINE . . . . 21<br>PIT LATRINE WITH SLAB . . . . 22<br>PIT LATRINE WITHOUT SLAB/<br>OPEN PIT . . . . 23<br>TWIN PIT/COMPOSTING TOILET . . . . . . 31<br>DRY TOILET . . . . 41<br>NO FACILITY/USES OPEN SPACE<br>OR FIELD . . . . 51<br>OTHER ________________96<br>(SPECIFY)	51 → 34
32	क्या इस शौच सुविधा का इस्तेमाल अन्य परिवार भी करते हैं?<br><br>Do you share this toilet facility with other households?	YES . . . . 1<br>NO 2	2 → 34

NO.	QUESTIONS AND FILTERS	CODING CATEGORIES	SKIP
33	कितने परिवार इस शौच सुविधा का इस्तेमाल करते हैं?<br><br>How many households use this toilet facility?	NO. OF HOUSEHOLDS IF LESS THAN 10 . . . . [0][ ]<br>10 OR MORE HOUSEHOLDS . . . . 95<br>DON'T KNOW 98	
34	परिवार के मुखिया का धर्म क्या है?<br><br>What is the religion of the head of the household?	HINDU . . . . 01<br>MUSLIM . . . . 02<br>CHRISTIAN . . . . 03<br>SIKH . . . . 04<br>BUDDHIST/NEO-BUDDHIST . . . . 05<br>JAIN . . . . 06<br>JEWISH . . . . 07<br>PARSI/ZOROASTRIAN . . . . 08<br>NO RELIGION . . . . 09<br>OTHER ______________________ 96<br>(SPECIFY)	
35	परिवार के मुखिया की जाति या जनजाति क्या है?<br><br>What is the caste or tribe of the head of the household?	CASTE ______________________ 991<br>(SPECIFY)<br>TRIBE ______________________ 992<br>(SPECIFY)<br>NO CASTE/TRIBE . . . . 993<br>DON'T KNOW 998	 <br><br><br>→ 37
36	क्या यह अनुसूचित जाति, अनुसूचित जनजाति, अन्य पिछड़े वर्ग में से है या इनमें से कोई नहीं है?<br>Is this a scheduled caste, a scheduled tribe, other backward class, or none of them?	SCHEDULED CASTE . . . . 1<br>SCHEDULED TRIBE . . . . 2<br>OTHER BACKWARD CLASS . . . . 3<br>NONE OF THEM . . . . 4<br>DON'T KNOW 8	

NO.	QUESTIONS AND FILTERS		CODING CATEGORIES	YES	NO	SKIP
37	क्या आपके घर में ___________हैं:<br>Does your household have:					
	a) Electricity?	बिजली?	ELECTRICITY	1	2	
	b) A mattress?	गददा?	MATTRESS	1	2	
	c) A pressure cooker?	प्रेशर कुकर?	PRESSURE COOKER	1	2	
	d) A chair?	कुर्सी?	CHAIR	1	2	
	e) A cot or bed?	खाट या चारपाई?	COT/BED	1	2	
	f) A table?	मेज?	TABLE	1	2	
	g) An electric fan?	बिजली का पंखा?	ELECTRIC FAN	1	2	
	h) A radio or transistor?	रेडियो या ट्रांजिस्टर?	RADIO/TRANSISTOR	1	2	
	i) A black and white television?	काला और सफेद टेलीविजन?	B & W TELEVISION	1	2	
	j) A colour television?	रंगीन टेलीविजन?	COLOUR TELEVISION	1	2	
	k) A sewing machine?	सिलाई मशीन?	SEWING MACHINE	1	2	
	l) A mobile telephone?	मोबाईल टेलीफोन?	MOBILE TELEPHONE	1	2	
	m) A land line telephone?	लैंडलाइन टेलीफोन?	LAND LINE TELEPHONE	1	2	
	n) Internet?	इंटरनेट?	INTERNET	1	2	
	o) A computer?	कम्प्युटर?	COMPUTER	1	2	
	p) A refrigerator?	रेफ्रिजरेटर?	REFRIGERATOR	1	2	
	q) An air conditioner/cooler?	ए सी / कूलर?	AIR CONDITIONER/COOLER .	1	2	
	r) A washing machine?	कपडे धोने की मशीन?	WASHING MACHINE	1	2	
	s) A watch or clock?	घड़ी या दीवार घड़ी?	WATCH/CLOCK	1	2	
	t) A bicycle?	साइकिल?	BICYCLE	1	2	
	u) A motorcycle or scooter?	मोटर साईकिल या स्कूटर?	MOTORCYCLE/SCOOTER	1	2	
	v) An animal-drawn cart?	जानवर द्वारा खींची जानेवाली गाड़ी?	ANIMAL-DRAWN CART	1	2	
	w) A car?	कार?	CAR	1	2	
	x) A water pump?	वाटर पंप?	WATER PUMP	1	2	
	y) A thresher?	थ्रेशर?	THRESHER	1	2	
	z) A tractor?	ट्रेक्टर?	TRACTOR	1	2	

NO.	QUESTIONS AND FILTERS	CODING CATEGORIES	SKIP
38	खाना पकाने के लिए आपके घर में मुख्यत: किस प्रकार के ईंधन का इस्तेमाल किया जाता है?<br>What type of fuel does your household mainly use for cooking?	ELECTRICITY . . . . 01<br>LPG/NATURAL GAS . . . . 02<br>BIOGAS . . . . 03<br>KEROSENE . . . . 04<br>COAL/LIGNITE . . . . 05<br>CHARCOAL . . . . 06<br>WOOD . . . . 07<br>STRAW/SHRUBS/GRASS . . . . 08<br>AGRICULTURAL CROP WASTE . . . . . . 09<br>DUNG CAKES . . . . 10<br>NO FOOD COOKED IN HOUSEHOLD . 95<br>OTHER __________ 96<br>(SPECIFY)	01–03 → 40<br>95 → 42
39	क्या इस घर में खाना स्टोव पर, चूल्हे पर या खुली आग में पकाया जाता है?<br>In this household, is food cooked on a stove, a chullah or an open fire?	STOVE . . . . 1<br>CHULLAH . . . . 2<br>OPEN FIRE . . . . 3<br>OTHER __________ 6<br>(SPECIFY)	
40	क्या खाना सामान्यतः घर में, अलग इमारत में या बाहर पकाया जाता है?<br>Is the cooking usually done in the house, in a separate building, or outdoors?	IN THE HOUSE . . . . 1<br>IN A SEPARATE BUILDING . . . . 2<br>OUTDOORS . . . . 3<br>OTHER __________ 6<br>(SPECIFY)	2, 3, 6 → 42
41	क्या आपके घर में खाना पकाने के लिए अलग कमरा है?<br>Do you have a separate room which is used as a kitchen?	YES . . . . 1<br>NO 2	
42	MAIN MATERIAL OF THE FLOOR.<br>RECORD OBSERVATION.	**NATURAL FLOOR**<br>MUD/CLAY/EARTH . . . . 11<br>SAND . . . . 12<br>DUNG . . . . 13<br>**RUDIMENTARY FLOOR**<br>RAW WOOD PLANKS . . . . 21<br>PALM/BAMBOO . . . . 22<br>BRICK . . . . 23<br>STONE . . . . 24<br>**FINISHED FLOOR**<br>PARQUET OR POLISHED WOOD . 31<br>VINYL OR ASPHALT . . . . 32<br>CERAMIC TILES . . . . 33<br>CEMENT . . . . 34<br>CARPET . . . . 35<br>POLISHED STONE/MARBLE/<br>GRANITE . . . . 36<br>OTHER __________ 96<br>(SPECIFY)	

NO.	QUESTIONS AND FILTERS	CODING CATEGORIES	SKIP
43	MAIN MATERIAL OF THE ROOF.<br><br>RECORD OBSERVATION.	**NATURAL ROOFING**<br>NO ROOF . . . . . 11<br>THATCH/PALM LEAF/<br>REED/GRASS . . . . . 12<br>MUD . . . . . 13<br>SOD/MUD AND GRASS MIXTURE . 14<br>PLASTIC/POLYTHENE SHEETING . 15<br>**RUDIMENTARY ROOFING**<br>RUSTIC MAT . . . . . 21<br>PALM/BAMBOO . . . . . 22<br>RAW WOOD PLANKS/TIMBER . . . . 23<br>UNBURNT BRICK . . . . . 24<br>LOOSELY PACKED STONE . . . . . . 25<br>**FINISHED ROOFING**<br>METAL/GI . . . . . 31<br>WOOD . . . . . 32<br>CALAMINE/CEMENT FIBER . . . . . 33<br>ASBESTOS SHEETS . . . . . 34<br>RCC/RBC/CEMENT/CONCRETE . . . . 35<br>ROOFING SHINGLES . . . . . 36<br>TILES . . . . . 37<br>SLATE . . . . . 38<br>BURNT BRICK . . . . . 39<br><br>OTHER ______________ 96<br>(SPECIFY)	
44	MAIN MATERIAL OF THE EXTERIOR WALLS.<br><br>RECORD OBSERVATION.	**NATURAL WALLS**<br>NO WALLS . . . . . 11<br>CANE/PALM/TRUNKS/BAMBOO . . . . 12<br>MUD . . . . . 13<br>GRASS/REEDS/THATCH . . . . . 14<br>**RUDIMENTARY WALLS**<br>BAMBOO WITH MUD . . . . . 21<br>STONE WITH MUD . . . . . 22<br>PLYWOOD . . . . . 23<br>CARDBOARD . . . . . 24<br>UNBURNT BRICK . . . . . 25<br>RAW WOOD/REUSED WOOD . . . . . . 26<br>**FINISHED WALLS**<br>CEMENT/CONCRETE . . . . . 31<br>STONE WITH LIME/CEMENT . . . . . 32<br>BURNT BRICKS . . . . . 33<br>CEMENT BLOCKS . . . . . 34<br>WOOD PLANKS/SHINGLES . . . . . . 35<br>GI/METAL/ASBESTOS SHEETS . . . . . . 36<br><br>OTHER ______________ 96<br>(SPECIFY)	
45	इस घर में सोने के लिए कितने कमरों का उपयोग किया जाता है?<br>How many rooms in this household are used for sleeping?	ROOMS . . . . . [ ][ ]	
46	क्या यह परिवार इस घर का या किसी दूसरे घर का मालिक है?<br>Does any member of this household own this house or any other house?	YES . . . . . 1<br>NO 2	 → 48
47	उस घर का मालिक कौन है?<br><br>Who owns the house?	MALE MEMBER . . . . . 1<br>FEMALE MEMBER . . . . . 2<br>BOTH . . . . . 3<br>DON'T KNOW 8	
48	क्या इस परिवार का कोई भी सदस्य किसी खेतीहर ज़मीन का मालिक है?<br>Does any member of this household own any agricultural land?	YES . . . . . 1<br>NO 2	 → 52

NO.	QUESTIONS AND FILTERS	CODING CATEGORIES	SKIP
49	इस खेतीहर ज़मीन का मालिक कौन है?<br><br>Who owns this agricultural land ?	MALE MEMBER . . . . 1<br>FEMALE MEMBER . . . . 2<br>BOTH . . . . 3<br>DON'T KNOW 8	
50	इस परिवार के सदस्यों के पास अपनी कितनी खेतीहर ज़मीन है?<br>How much agricultural land do members of this household own?<br><br>____________________<br>(IF NOT IN ACRES, SPECIFY SIZE AND UNIT)	ACRES . . . . . . [ ][ ][ ] . [ ]	
51	इस ज़मीन में से कितनी सिंचित है?<br><br>Out of this land, how much is irrigated?<br><br>____________________<br>(IF NOT IN ACRES, SPECIFY SIZE AND UNIT)	ACRES . . . . . . [ ][ ][ ] . [ ]<br><br>NONE . . . . 9995<br>DON'T KNOW 9998	
52	क्या आपके परिवार के पास इनमें से कोई मवेशी है:<br>Does your household own any of the following animals:<br><br>a) Cows, bulls, or buffaloes? गाय, बैल या भैंस?<br>b) Camels? ऊंट?<br>c) Horses, donkeys, or mules? घोडे,गधे या खच्चर?<br>d) Goats? बकरी?<br>e) Sheep? भेंड़?<br>f) Chickens or ducks? मुर्गा या बत्तख?	YES NO<br><br>COWS/BULLS/BUFFALOES . . . . 1 2<br>CAMELS . . . . 1 2<br>HORSES/DONKEYS/MULES . 1 2<br>GOATS . . . . 1 2<br>SHEEP . . . . 1 2<br>CHICKENS/DUCKS 1 2	
53	क्या इस परिवार के किसी सामान्य सदस्य का बैंक या डाकघर में कोई खाता है?<br>Does any usual member of this household have a bank account or a post office account?	YES . . . . 1<br>NO . . . . 2<br>DON'T KNOW 8	
54	क्या इस परिवार का कोई सामान्य सदस्य स्वास्थ्य योजना या स्वास्थ्य बीमा के अंर्तगत आता है?<br>Is any usual member of this household covered by a health scheme or health insurance?	YES . . . . 1<br>NO . . . . 2<br>DON'T KNOW 8	NO, DON'T KNOW → 56
55	स्वास्थ्य योजना या स्वास्थ्य बीमा किस प्रकार का है?<br>अन्य किसी प्रकार का?<br><br>What type of health scheme or health insurance?<br>Any other type?<br><br>RECORD ALL MENTIONED.	EMPLOYEES STATE<br>INSURANCE SCHEME (ESIS) . . . . . . A<br>CENTRAL GOVERNMENT HEALTH<br>SCHEME (CGHS) . . . . B<br>STATE HEALTH INSURANCE<br>SCHEME . . . . C<br>RASHTRIYA SWASTHYA BIMA YOJANA<br>(RSBY) . . . . D<br>COMMUNITY HEALTH INSURANCE<br>PROGRAMME . . . . E<br>OTHER HEALTH INSURANCE<br>THROUGH EMPLOYER . . . . F<br>MEDICAL REIMBURSEMENT FROM<br>EMPLOYER . . . . G<br>OTHER PRIVATELY PURCHASED<br>COMMERCIAL HEALTH INSURANCE . H<br>OTHER ________________ X<br>(SPECIFY)	

NO.	QUESTIONS AND FILTERS	CODING CATEGORIES	SKIP
56	जब आपके परिवार के सदस्य बीमार पड़ते हैं तो वे सामान्यतः इलाज के लिए कहां जाते हैं?<br><br>When members of your household get sick, where do they generally go for treatment?	**PUBLIC HEALTH SECTOR**<br>GOVT./MUNICIPAL HOSPITAL ...... 11<br>GOVT. DISPENSARY .... 12<br>UHC/UHP/UFWC .... 13<br>CHC/RURAL HOSPITAL/BLOCK PHC . 14<br>PHC / ADDITIONAL PHC .... 15<br>SUB-CENTRE .... 16<br>VAIDYA/HAKIM/HOMEOPATH (AYUSH) 17<br>ANGANWADI/ICDS CENTRE ...... 18<br>ASHA .... 19<br>GOVT. MOBILE CLINIC .... 20<br>OTHER PUBLIC SECTOR<br>HEALTH FACILITY .... 21<br><br>NGO OR TRUST HOSPITAL/CLINIC .... 31<br><br>**PRIVATE HEALTH SECTOR**<br>PVT. HOSPITAL .... 41<br>PVT. DOCTOR/CLINIC .... 42<br>PVT. PARAMEDIC .... 43<br>VAIDYA/HAKIM/HOMEOPATH (AYUSH) 44<br>TRADITIONAL HEALER .... 45<br>PHARMACY/DRUGSTORE .... 46<br>DAI (TBA) .... 47<br>OTHER PRIVATE SECTOR<br>HEALTH FACILITY .... 48<br><br>**OTHER**<br>SHOP .... 51<br>HOME TREATMENT .... 52<br><br>OTHER ______________ 96<br>(SPECIFY)	(11–18) → 58
57	आपके परिवार के सदस्य बीमार पड़ने पर सामान्यतः सरकारी सुविधा में क्यों नहीं जाते हैं?<br>कोई अन्य कारण?<br>Why don't members of your household generally go to a government facility when they are sick?<br>Any other reason?<br><br>RECORD ALL MENTIONED.	NO NEARBY FACILITY .... A<br>FACILITY TIMING NOT CONVENIENT .... B<br>HEALTH PERSONNEL OFTEN ABSENT · C<br>WAITING TIME TOO LONG .... D<br>POOR QUALITY OF CARE .... E<br><br>OTHER ______________ X<br>(SPECIFY)	
58	क्या इस परिवार के पास बी पी एल कार्ड है?<br><br>Does this household have a BPL card?	YES .... 1<br>NO .... 2<br>DON'T KNOW 8	
59	क्या इस परिवार के पास कोई मच्छरदानी है जिसका इस्तेमाल सोते समय किया जा सकता है?<br>Does your household have any mosquito nets that can be used while sleeping?	YES .... 1<br>NO 2	2 → 66
60	आपके घर में कितनी मच्छरदानियां हैं?<br>How many mosquito nets does your household have?<br>IF 7 OR MORE NETS, RECORD '7'.	NUMBER OF NETS .... [ ]	
61	मच्छरदानी (मच्छरदानियां) आपको कहां से मिली?<br><br>From where did you get the mosquito net(s)?<br><br>RECORD ALL MENTIONED.	PURCHASED FROM THE MARKET ...... A<br>GOVERNMENT .... B<br>SUPPLIED BY NGO/TRUST .... C<br><br>OTHER ______________ X<br>(SPECIFY)<br>DON'T KNOW .... Z	

		NET #1	NET #2	NET #3
62	OBSERVE OR ASK THE BRAND/ TYPE OF MOSQUITO NET.<br>IF BRAND IS UNKNOWN AND YOU CANNOT OBSERVE THE NET, SHOW PICTURES OF TYPICAL NET TYPES/BRANDS TO RESPONDENT.	**LONG-LASTING INSECTICIDE-TREATED NET (LLIN)**<br>DAWA PLUS . . . . 11<br>DURANET . . . . . . 12<br>INTERCEPTOR . 13<br>LIFENET . . . . . . 14<br>MAGNET . . . . . . 15<br>NETPROTECT . 16<br>OLYSET . . . . . . 17<br>PERMANET . . . . 18<br>ROYAL SENTRY . . 19<br>YORKOOL . . . . 20<br>OTHER/ DK BRAND . . . . 26<br><br>**'PRETREATED' NET** . 30<br><br>OTHER BRAND . . . . . . 96<br>DK BRAND 98	**LONG-LASTING INSECTICIDE-TREATED NET (LLIN)**<br>DAWA PLUS . . . . 11<br>DURANET . . . . . . 12<br>INTERCEPTOR . 13<br>LIFENET . . . . . . 14<br>MAGNET . . . . . . 15<br>NETPROTECT . 16<br>OLYSET . . . . . . 17<br>PERMANET . . . . 18<br>ROYAL SENTRY . . 19<br>YORKOOL . . . . 20<br>OTHER/ DK BRAND . . . . 26<br><br>**'PRETREATED' NET** . 30<br><br>OTHER BRAND . . . . . . 96<br>DK BRAND 98	**LONG-LASTING INSECTICIDE-TREATED NET (LLIN)**<br>DAWA PLUS . . . 11<br>DURANET . . . . . . 12<br>INTERCEPTOR . 13<br>LIFENET . . . . . . 14<br>MAGNET . . . . . . 15<br>NETPROTECT . 16<br>OLYSET . . . . . . 17<br>PERMANET . . . 18<br>ROYAL SENTRY . . 19<br>YORKOOL . . . 20<br>OTHER/ DK BRAND . . . 26<br><br>**'PRETREATED' NET** . 30<br><br>OTHER BRAND . . . . . . 96<br>DK BRAND 98
63	इस मच्छरदानी में पिछली रात को क्या क्या सोया था?<br>Did anyone sleep under this mosquito net last night?	YES . . . . 1<br>NO . . . . 2<br>(SKIP TO 65) ←<br>NOT SURE 8	YES . . . . 1<br>NO . . . . 2<br>(SKIP TO 65) ←<br>NOT SURE 8	YES . . . . 1<br>NO . . . . 2<br>(SKIP TO 65) ←<br>NOT SURE 8
64	इस मच्छरदानी में पिछली रात कौन सोया था?<br><br>Who slept under this mosquito net last night?<br><br>RECORD THE PERSON'S NAME AND LINE NUMBER FROM THE HOUSEHOLD SCHEDULE.	NAME ________<br>LINE NO. . . . . [ ][ ]<br>---<br>NAME ________<br>LINE NO. . . . . [ ][ ]<br>---<br>NAME ________<br>LINE NO. . . . . [ ][ ]<br>---<br>NAME ________<br>LINE NO. . . . . [ ][ ]	NAME ________<br>LINE NO. . . . . [ ][ ]<br>---<br>NAME ________<br>LINE NO. . . . . [ ][ ]<br>---<br>NAME ________<br>LINE NO. . . . . [ ][ ]<br>---<br>NAME ________<br>LINE NO. . . . . [ ][ ]	NAME ________<br>LINE NO. . . . . [ ][ ]<br>---<br>NAME ________<br>LINE NO. . . . . [ ][ ]<br>---<br>NAME ________<br>LINE NO. . . . . [ ][ ]<br>---<br>NAME ________<br>LINE NO. . . . . [ ][ ]
65		GO BACK TO 62 FOR NEXT NET; OR, IF NO MORE NETS, GO TO 66.	GO BACK TO 62 FOR NEXT NET; OR, IF NO MORE NETS, GO TO 66.	GO TO 62 IN FIRST COLUMN OF A NEW QUESTIONNAIRE; OR, IF NO MORE NETS, GO TO 66.

NO.	QUESTIONS AND FILTERS	CODING CATEGORIES	
66	कृपया मुझे वह स्थान दिखाएं जहां पर आपके घर के सदस्य प्रायः हाथ धोते हैं।<br><br>Please show me where members of your household most often wash their hands.	OBSERVED . . . .<br>NOT OBSERVED,<br>NOT IN DWELLING/YARD/PLOT . . . .<br>NOT OBSERVED,<br>NO PERMISSION TO SEE . . . .<br>NOT OBSERVED, OTHER REASON . . . .<br>(SKIP TO 69)	1<br><br>2<br><br>3<br>4
67	OBSERVATION ONLY:<br>OBSERVE PRESENCE OF WATER AT THE PLACE FOR HANDWASHING.	WATER IS AVAILABLE . . . .<br>WATER IS NOT AVAILABLE	1<br>2
68	OBSERVATION ONLY:<br>OBSERVE PRESENCE OF SOAP, DETERGENT, OR OTHER CLEANSING AGENT.	SOAP OR DETERGENT<br>(BAR, LIQUID, POWDER, PASTE) . . . .<br>ASH, MUD, SAND . . . .<br>NONE	 A<br>B<br>C
69	ASK RESPONDENT FOR A TEASPOONFUL OF COOKING SALT.<br><br>TEST SALT FOR IODINE.	IODINE PRESENT . . . .<br>NO IODINE . . . .<br><br>NO SALT IN HOUSEHOLD . . . .<br><br>SALT NOT TESTED ____________________<br>(SPECIFY REASON)	1<br>2<br><br>3<br><br>6

NO.	QUESTIONS AND FILTERS	CODING CATEGORIES	
70	January 2012 से लेकर क्या इस घर के किसी सामान्य सदस्य का मृत्यु हुआ है?<br><br>Did any usual member of this household die since January 2012?	YES . . . . 1<br>NO 2	 → END
71	कितने लोगों कि मृत्यु हुई है?<br>How many persons died?	TOTAL DEATHS . . . . [ ][ ]	

72<br>कृपया उनका नाम बताये जिनकी मृत्यु हुई.<br><br>Please tell the name(s) of the (person/ people) who died.	73<br>क्या (NAME) पुरुष था या स्त्री?<br><br>Was (NAME) male or female?	74<br>(NAME) कि आयु कितनी थी जब (उनका/उनकी) मृत्यु हुआ/हुई थीं?<br><br>How old was (NAME) when (he/she) died?	75<br>किस महिने और साल में (NAME) कि मृत्यु हुई?<br><br>In what month and year did (NAME) die?	76<br>क्या मृत्यु दुर्घटना, हिंसा, विष [जहर], हत्या या आत्महत्या के कारण हुई?<br><br>Was the death due to an accident, violence, poisoning, homicide or suicide?	**IF FEMALE AND DIED WHEN 12 YEARS OR OLDER:**<br>77<br>क्या (NAME) कि मृत्यु गर्भावस्था के दौरान, प्रसव के दौरान, या गर्भावस्था के समाप्ति या बच्चे के जन्म के दो महिने के अंदर हुई?<br><br>Did (NAME) die during pregnancy, during childbirth or within two months after the end of pregnancy or childbirth?
(1)<br><br>______<br>NAME	MALE . 1<br>FEMALE . 2	DAYS 1 [ ][ ]<br>MONTHS 2 [ ][ ]<br>YEARS 3 [ ][ ]	MONTH [ ][ ]<br>[ ][ ][ ][ ]<br>YEAR	YES . . . 1 → GO TO NEXT LINE<br>NO 2	YES . . . . 1<br>NO 2
(2)<br><br>______<br>NAME	MALE . 1<br>FEMALE . 2	DAYS 1 [ ][ ]<br>MONTHS 2 [ ][ ]<br>YEARS 3 [ ][ ]	MONTH [ ][ ]<br>[ ][ ][ ][ ]<br>YEAR	YES . . . 1 → GO TO NEXT LINE<br>NO 2	YES . . . . 1<br>NO 2
(3)<br><br>______<br>NAME	MALE . 1<br>FEMALE . 2	DAYS 1 [ ][ ]<br>MONTHS 2 [ ][ ]<br>YEARS 3 [ ][ ]	MONTH [ ][ ]<br>[ ][ ][ ][ ]<br>YEAR	YES . . . 1 → GO TO NEXT LINE<br>NO 2	YES . . . . 1<br>NO 2
(4)<br><br>______<br>NAME	MALE . 1<br>FEMALE . 2	DAYS 1 [ ][ ]<br>MONTHS 2 [ ][ ]<br>YEARS 3 [ ][ ]	MONTH [ ][ ]<br>[ ][ ][ ][ ]<br>YEAR	YES . . . 1 → GO TO NEXT LINE<br>NO 2	YES . . . . 1<br>NO 2
(5)<br><br>______<br>NAME	MALE . 1<br>FEMALE . 2	DAYS 1 [ ][ ]<br>MONTHS 2 [ ][ ]<br>YEARS 3 [ ][ ]	MONTH [ ][ ]<br>[ ][ ][ ][ ]<br>YEAR	YES . . . 1 → GO TO NEXT LINE<br>NO 2	YES . . . . 1<br>NO 2

INTERVIEWER'S OBSERVATIONS

TO BE FILLED IN AFTER COMPLETING INTERVIEW

COMMENTS ABOUT RESPONDENT:

COMMENTS ON SPECIFIC QUESTIONS:

ANY OTHER COMMENTS:

SUPERVISOR'S OBSERVATIONS

NAME OF SUPERVISOR: ______________________ DATE: ______________________
